# Repellency, Toxicity, Gene Expression Profiling and In Silico Studies to Explore Insecticidal Potential of *Melaleuca alternifolia* Essential Oil against *Myzus persicae*

**DOI:** 10.3390/toxins10110425

**Published:** 2018-10-25

**Authors:** Talha Ali Chohan, Tahir Ali Chohan, Lijun Zhou, Qianqian Yang, Liao Min, Haiqun Cao

**Affiliations:** 1 School of Plant Protection, Anhui Agricultural University, Hefei 230036, China; talhaali87@yahoo.com (T.A.C.); ljzhou@ahau.edu.cn (L.Z.); yangqianqian28@163.com (Q.Y.); jsxx@ahau.edu.cn (L.M.); 2 Institute of Pharmaceutical Sciences, University of Veterinary and Animal Sciences, Lahore 54000, Pakistan; tahir.chohan@uvas.edu.pk; 3 Provincial Key Laboratory for Agri-Food Safety, Hefei 230036, China

**Keywords:** aphid, essential oils, tea tree oil, pest control, docking, MD simulation

## Abstract

In the current study, deterrent assay, contact bioassay, lethal concentration (LC) analysis and gene expression analysis were performed to reveal the repellent or insecticidal potential of *M. alternifolia* oil against *M. persicae*. *M. alternifolia* oil demonstrated an excellent deterrence index (0.8) at 2 g/L after 48 h. The oil demonstrated a pronounced contact mortality rate (72%) at a dose of 4 g/L after 24 h. Probit analysis was performed to estimate LC-values of *M. alternifolia* oil (40%) against *M. persicae* (LC_30_ = 0.115 g/L and LC_50_ = 0.37 g/L respectively) after 24 h. Furthermore, to probe changes in gene expression due to *M. alternifolia* oil contact in *M. persicae*, the expression of *HSP 60*, *FPPS I, OSD, TOL and ANT* genes were examined at doses of LC_30_ and LC_50_. Four out of the five selected genes—*OSD*, *ANT, HSP 60* and *FPPS I*—showed upregulation at LC_50_, whereas, *TOL* gene showed maximum upregulation expression at LC_30_. Finally, the major components of *M. alternifolia* oil (terpinen-4-ol) were docked and MD simulated into the related proteins of the selected genes to explore ligand–protein modes of interactions and changes in gene expression. The results show that *M. alternifolia* oil has remarkable insecticidal and deterrent effects and also has the ability to affect the reproduction and development in *M. persicae* by binding to proteins.

## 1. Introduction

Pests, including weeds, pathogens and insects, are the tangible contestants of agricultural crops, and are responsible for 20–50% of total losses in crop production [1]. The most disastrous pests include Aphids (Hemiptera: Aphididae), which can be broadly classified into more than 4300 different species [2]. *M. persicae* (Green Peach Aphid) damage crops in three main ways, including through catering on crops, transmitting pathogenic viruses to plants, and by secreting honeydew, consequently leading to secondary fungal infection and suppression of photosynthesis [3]. *M. persicae* has become a serious threat to cultivation, causing tens of millions to billion-dollar losses in agriculture [4]. Several synthetic and semisynthetic insecticides have been developed and commercialized so far. However, decreased susceptibility and the development of huge resistance in *M. persicae* against multiple pesticides is another challenge for researchers. It has recently been reported to have developed resistance against at least seventy different commercially available pesticides [5]. Therefore, the prime attention of insecticide researchers has now been shifted towards identification and development of alternative green chemistries containing novel phytochemicals and biochemical targets implicated in pest control and resistance management [6]. Plant essential oils, as an emerging natural resource of insecticides, are considered to be broad spectrum and environmentally friendly, because the array of chemical constituents they contain quickly biodegrades in soil [7]. Moreover, the hydrophobic nature of essential oils may help them to interfere with the basic metabolic, biochemical, physiological and behavioral functions of insects [8]. Hence, identifying some plant-based essential oils with anti-insect potential against these resistant insects may represent an alternate and safer *M. persicae* management strategy.

The essential oils, mainly from the plants of the family Myrtaceae (Tea tree), represent a class of pharmacologically and toxicologically effective volatile secondary metabolites containing terpenes and aromatic compounds as major components [9]. *M. alternifolia* oil (Tea tree oil) is the essential oil extracted via distillation of the *Melaleuca alternifolia* (*M. alternifolia* or Tea Tree) leaves [10]. Despite remarkable pharmaceutical and therapeutic potential [11,12], *M. alternifolia* oil has also been known to possess repellent and insecticidal potential against a wide range insect pests [13]. Previous studies have shown that terpinen-4-ol, 1,8-cineole and terpinolene are the major constituents of the essential oils from *M. alternifolia* [14]. According to the International Standards Organization, ISO 4730 (ISO, 1996), terpinen-4-ol alone constitutes 30% of the total active components in essential oils from *M. alternifolia* [15]. Consequently, as a major component, terpinen-4-ol has been identified as a potential chemotype responsible for all of biological and anti-insect activities of *M. alternifolia* oil, and represents a novel botanical insecticide [11,12]. Although *M. alternifolia* has gained widespread recognition for its therapeutic and anti-insect potential, thus far, only limited published data on the insecticidal efficacy of edible tropical *M. alternifolia* essential oil against one of the most notorious pests (*M. persicae*) has been reported.

This study aims to investigate the repellent and insecticidal efficacy of *M. alternifolia* oils against *M. persicae*. A combination of deterrent assay, contact bioassay, and lethal concentration (LC) analysis has been performed to assess the deterrence and toxicity effects of *M. alternifolia* oil. We have also examined the effect of essential oil on the expression of five genes (*HSP 60*, *FPPS I*, *OSD*, *TOL* and *ANT*) in *M. persicae*. Subsequently, molecular docking and MD simulation were performed to predict binding mode of major chemotype (terpinen-4-ol) at the active site of selected gene-related proteins. The present study provides the detailed insight to the molecular events underlying reproductive, developmental and stress responses of *M. persicae* to *M. alternifolia* oil. The findings of this study may provide the foundation for developing novel environmentally friendly and biodegradable insecticide from edible plant essential oils.

## 2. Results and Discussion

**Figure 1:**
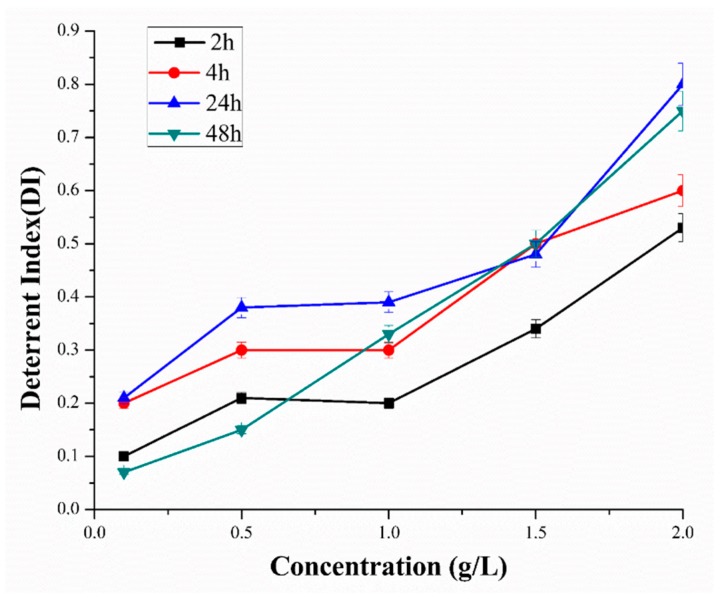
Deterrent activity of essential oils from *M. alternifolia* against *M. persicae* under choice conditions.

Essential oil from *M. alternifolia* has demonstrated great promise in pest management, and thus may represent an environmentally friendly alternative to conventional insecticides for *M. persicae* control. The phytochemical analysis (GC-MS) of *M. alternifolia* oil has revealed the presence of terpinen-4-ol (40.09%) and γ-terpinene (21.85%) as major components. In the present study, a 40% *M. alternifolia* oil formulation in five ascending concentrations (0.1–2 g/L distilled water) was used to investigate the deterrent effect of *M. alternifolia* oil against *M. persicae*. The essential oil from *M. alternifolia* has demonstrated promising ability to deter *M. persicae* under choice conditions. The highest deterrence index (DI) was observed at a 2 g/L concentration after exposure for 24 h and 48 h, with DI values of 0.8 and 0.75, respectively. These results suggest that the *M. alternifolia* oil has a dominant potential to repel *M. persicae* as compared to previously reported essential oils such as Pogostone oil (DI = 0.533 and 0.531 at 4 g/L, respectively, after 24 and 48 h) [16]. In addition, a similar level of repellency (DI: 0.5) against *M. persicae* was observed at a concentration of 1.5 mg/L of 40% *M. alternifolia* oil after 4, 24, or 48 h exposure (Figure 1). The overall results from the deterrent analysis have revealed that the *M. alternifolia* oil has fair to excellent repellency potential against *M. persicae* from low to high (0.1–2 g/L) concentration, respectively (Figure 1), which strongly suggests that *M. alternifolia* oil has sufficient deterrent potential to be applied in green houses.

**Figure 2:**
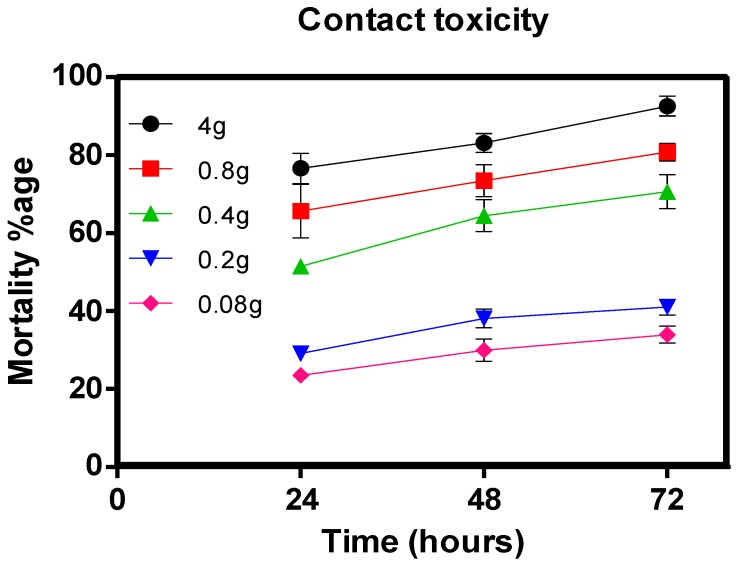
The percentage of contact toxicity at different time duration in response to different doses of the *M. alternifolia* oil, mortality was represented by percentage.

Furthermore, the contact toxicity of 40% essential oil from *M. alternifolia* was investigated against *M. persicae*, showing excellent mortality results at all doses, with a median LC_50_ value of 0.37 g/L after 24 h of exposure. Results suggest that *M. alternifolia* essential oil has greater insecticidal potential against *M. persicae* than previously reported essential oils from neem, eucalyptus, and laurel (LC_50_ = 0.5389, 0.9515, and 1.3730 g/L, respectively) [17]. At a higher dose (4 g/L), essential oil displayed the highest *M. persicae* mortality (76.61%) after 24 h, with further enhancement in mortality on the second day (48 h). Encouragingly, after 72 h treatment with the essential oil, exceptional results were obtained with a significant increase in *M. persicae* mortality (92.58%). The results clearly indicate that at the dose of 4 g/L, the mortality *M. persicae* significantly increases with time span (Figure 2). The LC_30_ and LC_50_ values were calculated by probit analysis as 0.115 g/L and 0.37 g/L, respectively, after 24 h of exposure to *M*. *alternifolia essential* oil.

### 2.1. Gene Expression

Gene expression analysis was performed in adult *M. persicae* to reveal the variation in gene expression upon prolonged exposure to two different sub-lethal concentrations (LC_30_, and LC_50_) of *M. alternifolia* oil. Gene expression analysis upon exposure to various treatments has previously been reported in terms of relative quantity (RQ) to that in controlled *M. persicae*, and at least 2-fold up- or downregulation was considered to be biologically significant [18]. In the present study, the variation in the expression of stress response (*HSP 60*), developmental (*FPPS I*) and dispersal (*OSD*, *TOL*, and *ANT*) genes [19] in adult *M. persicae* exposed to *M. alternifolia* oil for 24 h was studied.

#### 2.1.1. *OSD* Gene

**Figure 3:**
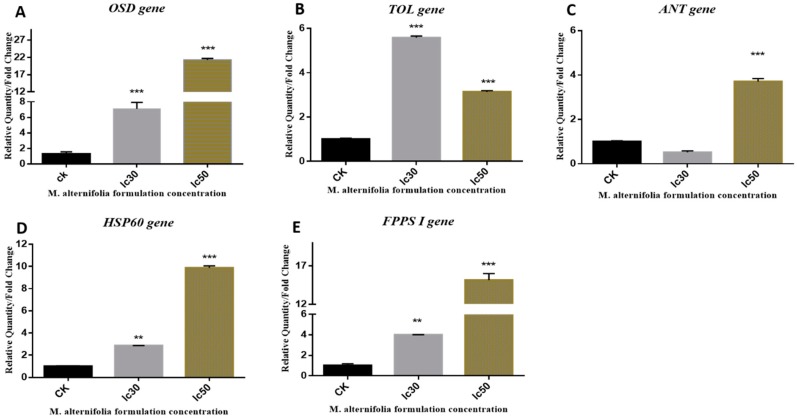
Relative gene expression of five different genes *OSD* (Olfactory Segment-D), *TOL* (Take-out like), *ANT* (Adenosine nucleotide translocase), *HSP 60* (Heat shock protein) and *FPPS I* (Farnesyl diphosphate synthase) were calculated at two lethal concentrations of *M. alternifolia* in adults *M. persicae* [20]. Mean values and standard deviations (SDs) are indicated by the error bar. *** Significant difference (*p* < 0.001), ** significant difference at *p* < 0.01.

A 21.26-fold upregulation of OSD gene expression was observed in adult *M. persicae* when exposed to a lethal dose (LC_50_) of 40% *M. alternifolia* oil (Figure 3A). The highest expression of *OSD* gene was observed at an LC_50_ dose of *M. alternifolia* oil, whereas a 14.15-folds lower *OSD* expression was observed at the LC_30_ dose (7.11) in adult *M. persicae* after the same time span.

#### 2.1.2. *TOL* Gene

Encouragingly, *M. alternifolia* essential oil induced significantly enhanced expression of *TOL* gene at both LC_30_ and LC_50_ doses. Surprisingly, the *TOL* gene demonstrated a different pattern of expression compared to the *OSD* gene upon exposure to *M. alternifolia* essential oil. The highest expression of the *TOL* gene was observed at the LC_30_ instead of the LC_50_ dose (Figure 3B).

#### 2.1.3. *ANT* Gene

In comparison to the *TOL* gene, enhanced expression of the *ANT* gene was observed in adult *M. persicae* on exposure to *M. alternifolia* oil (LC_50_) after 24 h (Figure 3C). Significant *ANT* gene expression was observed at LC_50_ dose *of M*. *alternifolia* oil, but no significant change was observed at LC_30_. The trend of *ANT* expression against different concentrations of *M. alternifolia* oil was also observed to be different from the *TOL* gene (Figure 3C).

*OSD*, *TOL* and *ANT* genes, also known as dispersal-related genes, have been reported to be overexpressed in *M. persicae* in response to stress. In the present work, all three genes exhibited upregulation; however, *OSD* has demonstrated a several-fold amplified upregulation in *M. persicae* in response to an LC_50_ dose of *M. alternifolia* oil. Accumulated evidence [21] indicates that elevated levels of *OSD* in *M. persicae* may suppress fecundity, which ultimately corresponds to decreased reproduction. Alternatively, the *ANT* gene essentially regulates mitochondrial proteins that act as carriers of important metabolites responsible for mediating several mitochondrial functions, i.e., catalyzing trans-membranous (mitochondrial) transport of ADP for synthesis of ATP [22]. The moderate response of the *ANT* gene to an LC_50_ dose of *M. alternifolia* oil indicates negligible additional energy expenditures for *M. persicae*. Meanwhile, unresponsiveness of the *ANT* gene against an LC_30_ dose, suggests that exposure to low concentrations of *M. alternifolia* oil results in no additional energy expenditures for the *M. persicae. TOL* can be overexpressed in response to starvation [23,24] and fluctuation in JH (Juvenile hormones) titers [25] during courtship and mating [26]. During gene expression analysis, the *TOL* gene has displayed quite different regulation trends in comparison to the *OSD* gene. Elevation of *TOL* gene expression was observed at LC_30_ and LC_50_ doses. At an LC_30_ dose of 40% *M. alternifolia* oil, substantial upregulation was observed in the *TOL* gene. Previously, reported upregulation of *TOL* gene has indicated that juvenile hormones binding proteins due to fluctuation of JH titers can cause starvation, affect antennal responses to food.

#### 2.1.4. *HSP 60* Gene

*HSP 60* belongs to the stress-response class of genes, as its expression has been demonstrated to vary according to stressor type. A plethora of studies has clearly demonstrated that downregulation of *HSP* (*HSP 60*, *HSP 70*, and *HSP 90*) expression is associated with a recovery response to regain homeostasis following prolonged exposure to mild levels of stress. Conversely, the upregulation of *HSP 60* reflects the accumulation of damaged proteins in response to stress or injury in an organism [27]. In addition, *HSP* accumulation has been reported to decrease fecundity [28]. In the present study, following topical treatment of adult *M. persicae* with 40% *M. alternifolia* oil, an almost 9.87-fold increased expression of the *HSP 60* gene was observed at an LC_50_ dose after 24 h exposure (Figure 3D). Meanwhile, the least response was observed against the LC_30_ dose. Comparing our results with previous studies, it can be speculated that *M. alternifolia* oil at higher doses negatively influences *M. persicae* reproduction by suppressing fecundity.

#### 2.1.5. *FPPS I* Gene

Enhanced expression of the *FPPS I* gene was observed in adult *M. persicae* at both concentrations (LC_30_ and LC_50_) of *M. alternifolia* oil (Figure 3E). At the highest concentration, LC_50_ of 40% *M. alternifolia* oil, the highest (15.155) upregulation in the *FPPS I* gene was observed. *M. alternifolia* oil at LC_30_ displayed 4-fold upregulation in *FPPS I* gene expression (Figure 3E). Previously, *FPPS I* has been shown to influence JH biosynthesis by catalyzing the formation of farnesyl diphosphate (FPP). JH has been reported to stimulate reproduction and sexual pheromones in *M. persicae*; meanwhile, elevated levels of JH titers in female *M. persicae* promote development of apterous forms by inhibiting wing development. Moreover, *FPPS I* downregulation is also linked to decreased (E) β-farnesene (EBF) production [29,30], which may increase fecundity in *M. persicae*. In short, the previous studies have demonstrated that *FPPS I* gene expression is inversely related to fecundity and reproduction in *M. persicae*, thus suggesting that an increase in *FPPS I* expression may negatively influence fecundity and reproduction in *M. persicae*. In the present study, *M. alternifolia* oil was observed to significantly upregulate *FPPS I* gene expression in adult *M. persicae* at both concentrations after 24 h. These findings propose that the phytochemical constituents of *M. alternifolia* oil negatively affect the fecundity and reproduction of *M. persicae*.

### 2.2. In Silico Studies

In silico investigation of structural and functional relationship may provide substantial knowledge about molecular mechanism for variations in target gene expression at the genome level. In silico approaches have also been applied previously to reveal the effect of different agonists or antagonists on the expression of their targeted genes [31,32]. During gene expression analysis, the expression of *OSD*, *FPPS I* and *HSP 60* was observed to be significantly upregulated upon continuous exposure to *M. alternifolia* oil. Knowledge of the 3D structures of *OSD*, *FPPS I* and *HSP 60* is mandatory to understand proteins interactions, functions and their binding residues. Herein, molecular modeling with subsequent docking and MD simulations were performed to investigate the molecular bases that regulate the expression of *M. persicae* proteins upon exposure to the major components of essential oil.

#### 2.2.1. Structural Description of the *OSD*, *FPPS I*, and *HSP 60* 3D Model

In this study, structure-based sequence analysis was performed on three proteins *OSD*, *FPPS I*, and *HSP 60*. The protein sequences were obtained using accession numbers (Table S2 of Supporting Information) from the NCBI protein. The primary structure analysis showed that the *OSD*, *FPPS I*, and *HSP 60* proteins had molecular weights of 15.03, 45.35 and 60.47 kilo-Daltons, respectively. The theoretical isoelectric points (pI) were calculated as 7.54, 5.25, and 6.81 for *OSD*, *FPPS I*, and *HSP 60*, respectively, indicating that the proteins were stable. The negative grand average of hydropathicity (GRAVY) showed values of −0.506, −0.046, and −0.116, indicating that the *OSD* protein exhibited higher hydrophilicity than *FPPS I* and *HSP 60* [33]. The sequence and secondary structure analyses results, summarized in Table S2 of the Supporting Information, reveal that all three protein structures are predominated by α-helix (55–75%). The secondary structure of *HSP 60* is composed of 12% β-plated sheets, while in *OSD* and *FPPS I*, no β-plated sheets were identified. Secondary structural features are shown in Figure S1A–C of the Supporting Information. Finally, the 3D structures of *OSD*, *FPPS I*, and *HSP 60* proteins (Figure S2A–C of the Supporting Information) were predicted using the online server *I-TASSER* and the best predicted structure with the maximum confidence score (*C*-score = *OSD* (−1.28), *FPPS I* (−1.18), and *HSP 60* (−0.25)) was selected to proceed towards ligand–protein docking studies. The quality and reliability of these protein structures was assessed using a Z-score and Ramachandran plot (Figure S2D–F of the Supporting Information). The Ramachandran plot revealed that 91.5%, 96.9% and 87.7% of residues were in the favorable region for the *OSD*, *FPPS I* and *HSP 60* proteins, respectively. Moreover, the model quality was further checked against the Z-score by using the input structure in the range of scores typically found for native proteins of similar size. The best predicted structure with the maximum confidence score (*C*-score) and Z-score (Table S2 of Supporting Information) were selected for further simulation and analysis.

#### 2.2.2. Molecular Docking and Molecular Dynamics Simulation

In the present work, two major components (terpinen-4-ol and γ-terpinene) of *M. alternifolia* oil were docked in the active sites of *OSD*, *FPPS I*, and *HSP 60* proteins using the Surflex-Dock module of *SYBYL-X 1.3* [34]. The docking scores (*C*-score) of terpinen-4-ol for *OSD,* *FPPS I* and *HSP 60* proteins are 5.23 and 4.55 and 4.42, respectively, which indicates that terpinen-4-ol exhibits slightly higher binding affinity towards *OSD* than towards the *FPPS I* and *HSP 60* proteins. However, γ-terpinene displayed relatively weaker binding affinities towards all three selected proteins (*Cscore* = 2.22 (*OSD*), 2.55 (*FPPS I*) and 3.11 for *HSP 60*). To provide a more detailed insight into the ligand–protein interactions, the binding energies (consensus scores) and key hydrogen bond interactions are tabularized in Table S3 of supplementary information.

**Figure 4:**
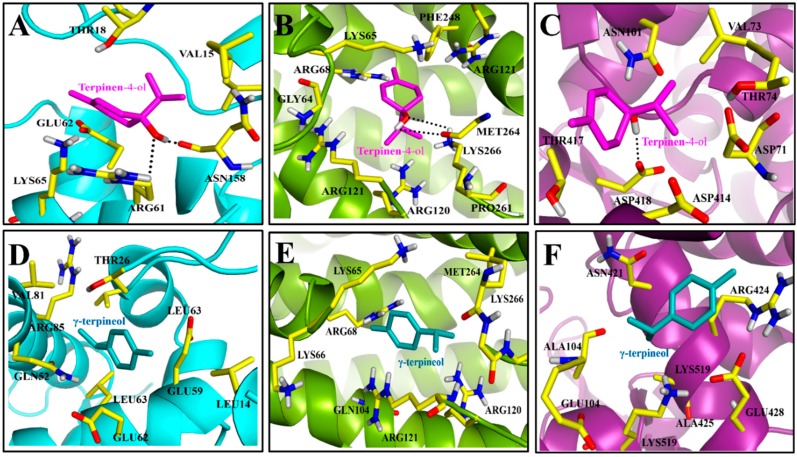
Graphical view of molecular docking results. The binding modes of ligands terpinen-4-ol (**A**–**C**) and γ-terpineol (**D**–**F**) within the active sites of the *OSD* (cyan), *FPPS I* (green) and *HSP 60* (Magenta) proteins, respectively. H-bonds are indicated as a dotted line.

Furthermore, the top-ranked docking poses for both ligands in all six complexes were saved and graphically viewed to identify the ligand–protein interactions. The graphical analysis shows that both ligands acquire a similar mode of interaction within the active site of the *OSD* protein and Val15, Thr18, Arg61, Glu62, Lys65, and Asn158 are the most important residues present at the active site. As depicted in Figure 4A,D, despite similar binding modes, terpinen-4-ol can form at least two H-bond interactions with surrounding residues; whereas, no H-bond interaction was observed in the γ-terpineol-*OSD* complex. The docked model of terpinen-4-ol reveals that the -OH groups of compound were able to form H-bond interactions following a donor–accepter motif with Asn158 and Arg61, respectively. These findings can explain the higher binding affinity of terpinen-4-ol towards the *OSD* protein than γ-terpineol.

In *FPPS I*-terpinen-4-ol and γ-terpineol docking complexes, the residues Gly64, Lys65, Arg68, Arg120, Arg121, Phe248, Pro261, Met264, Lys266, and Arg360 have been identified as the active site residues (Figure 4B,E). Docking results show that two residues, Met264 and Lys266, are able to establish H-bond interaction with the -OH group of terpinen-4-ol. The methyl groups extend towards Lys65, Arg68, and Phe248, where they can make van der Waals and hydrophobic contacts with these residues. Alternatively, no hydrogen bond interaction was observed in γ-terpineol, because it lacks heavy atoms in its structure.

Finally, to elucidate the interaction mode of both ligands with HPS-60, the top ranked docked poses of terpinen-4-ol and γ-terpineol in *HPS 60* active site were also visualized (Figure 4C,F). The docking results show that almost all residues that contribute towards terpinen-4-ol binding to *HSP 60* also have favorable interactions in γ-terpineol-*HSP 60* complex. However, the difference in binding affinity was mainly mediated by additional H-bond interaction formed between the -OH group of terpinen-4-ol and the nearby residue Asp418 in the binding pocket of *HSP 60*. Whereas, no H-bond interaction was found in the case of γ-terpineol-*HSP 60* system. Hence, molecular docking results provide sufficient information to identify that the difference in protein expression is mainly mediated by terpinen-4-ol, rather than any other compound. However, to further explore the binding affinity of terpinen-4-ol towards *OSD*, *FPPS I* or *HSP 60* proteins, molecular dynamics (MD) simulations were performed, followed by free energy calculations.

#### 2.2.3. Molecular Dynamics Simulation, MMGB/PBSA

**Figure 5:**
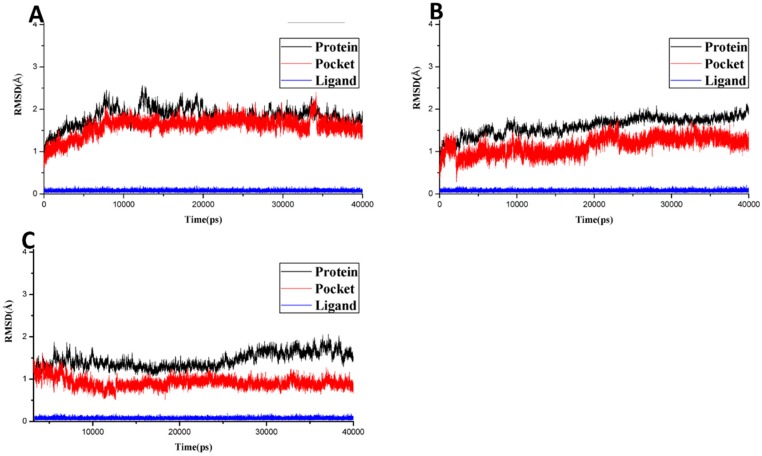
RMSDs of Cα atoms of the protein, backbone atoms of binding pocket (within 6.5 Å), and the heavy atoms in the ligand for: (**A**) terpinen-4-ol-*OSD* (**B**) terpinen-4-ol-*FPPS I*, and (**C**) terpinen-4-ol-*HSP 60*.

Although docking analysis can provide an acceptable binding mode, the solvent, temperature and pressure effects could not be considered. Therefore, docking results were post-processed with more reliable molecular dynamics (MD) simulations to describe the dynamical behavior of the complex at an atomic level by flexibly and firmly treating the ligand–receptor complex. Moreover, it also makes it possible to calculate the binding free energies using an implicit MMGB (PB) SA approach, which provides an accurate ranking of potential ligands binding to the target protein. The top three docking complexes (terpinen-4-ol-*OSD*, terpinen-4-ol-*FPPS I*, and terpinen-4-ol-*HSP 60*) as ranked by *C*-score were further processed using MD simulations to investigate the key molecular interactions responsible for ligand–receptor binding. All three complexes were subjected to 40 ns MD simulations, and RMSD (root-mean-square deviation) was calculated to inspect the dynamic stability of all complexes during simulation. As illustrated in Figure 5A–C, all complex systems remained stable and the RMSD remained below 2.5 Å for protein, pocket or ligand throughout the simulation. The least RMSD was found to be displayed by the ligand (blue) in all three complexes. However, this was not surprising, because terpinen-4-ol exhibited a smaller structure with very low flexibility. Overall, these analyses for ligand–protein complex stabilization suggest that the simulated docking conformations are correct and can be used to calculate binding free energies.

**Figure 6:**
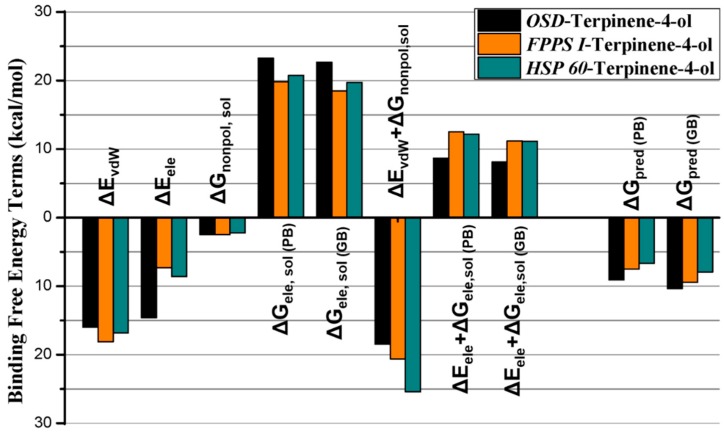
Comparison between binding free energy terms of (**A**) terpinen-4-ol-*OSD*, (**B**) terpinen-4-ol-*FPPS I*, (**C**) terpinen-4-ol-*HSP 60*.

The binding free energies of terpinen-4-ol bonded to *OSD*, *FPPS I*, and *HSP 60* were computed using the MMGB/PBSA approach. The results are plotted and summarized in Figure 6, and Table S3, in the Supporting Information, depicts a comparison between the binding free energy components in all three complexes. The results demonstrate a noticeable difference in the computed binding affinities for compound terpinen-4-ol against *OSD* (Δ*G*_pred (GB)_ = −10.34 kcal/mol, *FPPS I* (Δ*G*_pred (GB)_ = −9.43 kcal/mol) and *HSP 60* (Δ*G*_pred (GB)_ = −7.94 kcal/mol). Although the difference in binding free energies is not very high, it sufficiently reflects the fact that terpinen-4-ol exhibits greater binding affinity towards *OSD* than *FPPS I* and *HSP 60*. Interestingly, these results are also in agreement with molecular docking and experimental gene expression analysis. To identify the key driving forces responsible for the higher binding affinities of terpinen-4-ol, total binding free energy was further decomposed into independent binding free energy components (Figure 6 and Table S4). As shown in Figure 6, although van der Waals (vdW) interactions and nonpolar solvation display a slight favorable contribution towards *FPPS I*, the electrostatic force play substantial role in the higher binding affinity of terpinen-4-ol towards *OSD* than towards *FPPS I* and *HSP 60*. In the *HSP 60*-terpinen-4-ol complex system, the vdW forces display an almost comparable contribution for *OSD* and *HSP 60*, while the major difference in binding affinity arises from electrostatic interaction. These results are consistent with the results obtained from GRAVY analysis, which revealed that *OSD* is more hydrophilic than *FPPS I* and *HSP 60*. Moreover, according to the molecular docking results for the *HSP 60*-terpinen-4-ol system, there was only one H-bond interaction, as compared to two H-bond interaction in *OSD*. These results also support the MD simulation results. Hence, it might be speculated that the higher binding affinities of inhibitor terpinen-4-ol is mainly dominated by electrostatic and polar solvation free energies along with moderate vdW and H-bond interactions. These findings may further be used to identify some novel compounds with promising potential to interact with developmental and dispersal-related genes and may ultimately present an alternative strategy in *M. persicae* management.

## 3. Significance of Study

*M. alternifolia* oil has already been proved to exhibit promising insecticidal potential against various insects. However, no studies have been reported regarding its insecticidal potential against *M. persicae*. In this work, we studied the deterrent and toxicity potential of *M. alternifolia* essential oil against *M. persicae* for the first time. To study the impact of essential oil, a combination of gene expression analysis with molecular docking and MD simulations was also performed for the first time. As a result, *M. alternifolia* oil has been identified to possess significant deterrent and toxic potential against *M. persicae. M. alternifolia* oil has significantly enhanced upregulation of *OSD*, *TOL*, *ANT*, *HSP 60* and *FPPS I* genes, which negatively influence the fecundity, growth and development of *M. persicae*. An exquisite combination of molecular docking, MD simulation and binding free energy calculation (MMGB/PBSA) was performed to reveal the binding modes of terpinen 4-ol and γ-terpinene in complex with *FPPS I*, *HSP 60* and *OSD* genes. The graphical and energetic analysis revealed that terpinen-4-ol develops more favorable H-bond and hydrophobic interactions with the surrounding residues. The findings of the present study provide valuable guidelines for identifying a better ecofriendly alternative to conventional insecticides.

## 4. Conclusions

In the present study, *M. alternifolia* essential oil has displayed mild to excellent (concentration dependent) anti-*M. persicae* potential, due to its promising deterrent and insecticidal activities. The significant outcomes in biological and in silico studies are likely a result of the amalgamation of various secondary metabolites constituting the essential oil. Gene expression assays, along with in silico studies, identified terpinen-4-ol as a key component, responsible for significant deterrent and insecticidal activities of *M. alternifolia* essential oil, since in the present work, the essential oil from *M. alternifolia* alone showed appreciable insecticidal potential. Furthermore, research focused on combination of *M. alternifolia* essential oil with other oils or combination of terpinen-4-ol with other chemo-types against *M. persicae* is extensively required. The findings of the present study could provide valuable guidelines for the rational design and identification of novel compounds either in lab or from natural sources, respectively, with better insecticidal properties and a high safety profile.

## 5. Materials and Methods

### 5.1. 40% M. alternifolia Essential Oil Preparation

Essential oil of *M. alternifolia* was sourced from Fujian Senmeida Biological Technology Co., Ltd. (Fuzhou, Fujian, China). The insecticidal properties of essential oils are primarily attributed to the blend of various major and minor phytochemicals. Content assessment analysis for ten major components in the *M. alternifolia* were predetermined by GC/MS and reported in our previous study [35], which indicated that the major constituents terpinen-4-ol (40.09%), γ-terpinene (21.85%), α-terpinene (11.34%), α-terpineol (6.91%), α-pinene (5.86%), terpinolene (3.24%), 1,8-cineole (1.83%) limonene (1.20%), p-cymene (1.36%), and sabinene (0.20%) were within the range specified according to Inter-national Organization for Standardization standard 4730.

The formulation of 40% *M. alternifolia* essential oil was prepared by the general method [36]. The emulsifiers used for dispersing essential oil in water, thickening agents, and anti-freezing agents used in the formulation, along with their quantities (*g*/*g*), is tabularized in Table 1.

### 5.2. Myzus Persicae Culture

The susceptible laboratory population of *M. persicae* (Sulzer) used in this study was originally obtained from cabbage on the campus of Anhui Agriculture University. Then it was maintained in our laboratory at Anhui Agriculture University under 23 ± 2 °C, 75 ± 5% RH, and a photoperiod of L12:D12. *M. persicae* was reared on the insecticide-free cabbage seedlings. After ten generations in our insectary, 1-day-old apterous *M. persicae* were used for the further experiment.

### 5.3. Bioassay of M. persicae

#### 5.3.1. Deterrent Assay

The area preference method [37] was adopted to estimate the repellency potential of the *M. alternifolia* oil in five gradually increasing concentrations (0.1, 0.5, 1, 1.5, and 2 g/L). A filter paper disk of 9 cm in diameter was cut into semicircles. One half was dipped into the sample solutions while the second half was treated with water (controlled) for a time of 30 s. Both parts of filter papers were air dried at room temperature and placed at the bottom of petri dishes of the same size with connected edges. Twenty-five adult *M. persicae* were introduced at the center of petri dish containing filter paper and the petri dish was covered with a para film and lid to avoid the insects’ escape. Each treatment was replicated three times and the numbers of insects present on the control- and the sample-treated halves were recorded after 2, 4, 24 and 48 h. All petri-dishes were then placed in controlled conditions. A deterrence index was calculated for each dish as follows:

(1) $$\mathrm{Detterence}\;\mathrm{index}=\frac{\left(C-T\right)}{\left(C+T\right)}$$

where C is the number of *M. persicae* on the controlled half and T is the number of *M. persicae* on the treated paper after 2, 4, 24 and 48 h.

#### 5.3.2. Leaf-Dip Bioassay

The leaf dipping method was used to evaluate the toxicity of the *M. alternifolia* oil in the petri dish [38]. The test solution of 40% *M. alternifolia* oil was further diluted with distilled water containing 0.05% (*v*/*v*) Triton X-100, to prepare the following serial concentrations: 0.08, 0.2, 0.4, 0.8 and 4 g/L. Cabbage leaf discs 20 mm in diameter were prepared and immersed in the prepared solution for 30 s. The leaf discs were shed dried by exposing them to air for 2 h, and then placed upside down in a petri dish filled with 2% agar. The control was dipped into distilled water containing 0.05% (*v*/*v*) Triton X-100 and shed dried by exposure to air. Twenty-five adult healthy *M. persicae* were carefully transferred on the surface of the leaf with the help of soft brush. All discs were critically examined to identify and immediately remove sick or molting *M. persicae*. Each petri dish, both for the sample and the control, was covered with para film. All treatments were replicated at least three times, and the rate of mortality was recorded at 24, 48, and 72 h post treatment. The *M. persicae* were considered dead when they did not move any of their legs after probing with a soft brush. LC_30_ and LC_50_ were calculated by prohibit analysis [39]. The percentage of dead *M. persicae* was calculated by the Abbott’s formula [40]. The mortalities of all of the control were lower than 5%.

### 5.4. Real-Time Quantitative PCR (qRT-PCR)

To investigate the expression levels of five candidate genes under different concentrations (LC_30_ and LC_50_ values at 24 h) of 40% *M. alternifolia* oil, qRT-PCR was employed. Under each concentration, at least 40 *M. persicae* were collected at 24 h post-treatment, and immediately placed in liquid nitrogen to be stored at −80 °C until analysis. Firstly, total RNA was extracted from each treatment using TRIZOL reagent (Invitrogen, Carlsbad, MX, USA.) according to the manufacturer’s instructions. RNA integrity was evaluated and quantized by Biophotometer Plus (Eppendorf, Hamburg, Germany). First-strand complementary DNA (cDNA) was synthesized using Prime Script™ reagent kit (Takara, Dalian, China) in accordance with the manufacturer’s instructions. For qRT-PCR, *β-actin* was used as internal control for the quantification of *OSD* and *TOL*, and the *ACE* gene for *ANT*, *HSP 60*, and *FPPS I*. All primers for qRT-PCR are listed in Table S1. qRT-PCR was performed on a Bio-Rad iCycler iQ Real-time Detection System (Bio-Rad, Hercules, CA, USA.) containing 7.5 μL of 2× UltraSYBR Mixture (Promega Corporation, Beijing, China), 2 μL of cDNA, 1 μL each primer (10 μM), and 3.5 μL of RNase-free water in a final volume of 15 μL. In all qRT-PCR, each treatment was repeated three times. The gene expression (mean ± SD) quantified as a relative fold change was performed using the 2^−ΔΔCT^ method [41]. To determine the relative quantitative fold expression change, data was analyzed by ordinary one-way ANOVA of the *t*-test, and their compression between doses was analyzed by Tukey’s test [20].

### 5.5. Computation Methods

#### 5.5.1. Sequence Analysis and Modeling

To the best of our knowledge, the co-crystalized 3D structures for *OSD*, *FPPS I* and *HSP 60* proteins of *M. persicae* have not been reported to the Protein Data Bank (PDB) yet. The three-dimensional structure was predicted by the online server iterative threading assembly refinement algorithm implemented in I-TASSER. The FASTA sequences with NCBI Accession no # CAB58441, CAI34909 and XP_022183539 for *HSP 60*, *OSD* and *FPPS I*, respectively, were retrieved from uniprot [42,43], and submitted to the I-TASSER online server [42] for protein modeling. The I-TASSER-generated and -optimized 3D-models of the selected proteins were downloaded along with their confidence score (*C*-score). Structural and stereo chemical analyses were performed by using various evaluations and validation tools after generating 3D model. PROCHECK was used to attain the Psi/Phi Ramachandran plot [44], which can be utilized to assess the non-Gly residues in the disallowed regions. The ProSAweb web tool [45] employs an empirically derived Z-score functionality that was used to ensure the thorough quality of generated structural model and certify that the anticipated structure is within the range of scores as recovered in the native proteins. Only the top-ranking generated conformations according to the Z-score of each protein were further processed for molecular docking and MD simulations. Finally, the generated structures were energy-minimized and MD-simulated for 10 ns, which will be discussed in a later section.

#### 5.5.2. Ligand Preparation and Molecular Docking

The 3D structures of the terpinen-4-ol and γ-terpinene were constructed by the Sybyl-X1.3/SKETCH module [46], and energy was minimized using the Tripos force field with Gasteigere Hückel atomic charge. Both structures were further subjected to the MD approach for further optimization to obtain active geometrical conformation. To reveal the binding modes of selected ligands bonded with respective proteins, flexible molecular docking simulations were performed using the Surflex-Dock module of the molecular modeling software package *SYBYL-X 1.3* [34,47]. First of all, to ensure chemical accuracy, structures of all three *M. persicae* proteins were carefully examined by adopting structure preparation tools applicable in the biopolymer module of *SYBYL-X 1.3* [46]. Missing hydrogens were added, charges were applied, and atom types were assigned in accordance to the AMBER 7 FF99 force field. At the end, energy minimization was executed to hamper steric clashes by utilizing the Powell algorithm along with a convergence gradient of 0.5 kcal/(mol·Å) for 1000 cycles. Surflex-docking utilized an idealized active site ligand known as protomol [34], which provides a target site to generate putative poses of small molecules to guide the molecular docking. The parameters for protomol generation, such as threshold (0.50) and bloat (zero), were kept at default values. These settings are the same as those used in our previously reported docking validation studies [48,49]. Finally, the generated and energy-optimized conformations of terpinen-4-ol and γ-terpinene were separately docked to the active sites of *OSD*, *FPPS I* and *HSP 60*. For each ligand–protein complex system, the twenty best docked poses were saved conclusively for every inhibitor. By adopting the Hammerhead scoring function, these putative poses of ligands were graded [34].

#### 5.5.3. Molecular Dynamics Simulations

For further refinement and stabilization in solution systems, each of the top ranked docking-simulated structural model (terpinen-4-ol-*OSD*, terpinen-4-ol- *FPPS I* and terpinen-4-ol-*HSP 60*) complexes was subjected to MD simulations for 40 nanoseconds (ns). All MD simulations and molecular mechanics-based free energy calculations (MM/PB(GB)SA) [50], were entirely carried out in the AMBER16 software package [51], following the same protocol and parameters as those reported in our previous publications [48,49,52].

## Figures and Tables

**Table 1:** List of the main components of 40% *M. alternifolia* essential oil.

Compound	% (*g*/*g*)	Properties
*M. alternifolia* essential oil	40	Active ingredient
Ethylene glycol	2.5	Antifreeze
Epoxy ethane epoxide propane block polymer	2.5	Emulsifier
Polyoxyethylene Polyoxypropylene ether	2.5	Emulsifier
Silicone	0.3	Emulsifier
Gelatin	0.2	Thickener
Water	52	Deionized water

## Supplementary Materials

The following are available online at http://www.mdpi.com/2072-6651/10/11/425/s1, Figure S1: Secondary structures of (A) *OSD*, (B) *FPPS 1* (C) *HSP-60* predicted with Phyre2 online webserver, Figure S2: 3D structures of proteins built by I-TASSER. (A) *OSD*, (B) *FPPS 1* (C) *HSP-60*. Ramachandran Plot analysis performed with RAMPAGE online webserver. (D) *OSD*, (E) *FPPS 1* (F) *HSP-60*, Table S1: List of the selected primers (forward and reverse) which used for the measurements of gene expression in the *M. persicae* at three different lethal concentration of the *M. alternifolia* oil, Table S2: summary of pre- and post-molecular modeling analysis, Table S3: Surflex score of docked ligands terpinen-4-ol and γ-terpinene for *OSD*, *FPPS 1*, and *HPSA-60*, Table S4: Comparison between binding free energies of terpinen-4-ol bonded with *OSD*, *FPPS I*, *HSP 60*.
