# Supplementary material for: Repellency, Toxicity, Gene Expression Profiling and In Silico Studies to Explore Insecticidal Potential of Melaleuca alternifolia Essential Oil against Myzus persicae
Source: Toxins (Basel). 2018 Oct 25;10(11):425. doi: 10.3390/toxins10110425 (PMC6266121; doi:10.3390/toxins10110425)
Supplement: Supplementary file 1 [file toxins-10-00425-s001.pdf]

Supplementary Materials: Repellency, Toxicity, Gene Expression Profiling and In Silico Studies to Explore Insecticidal Potential of *Melaleuca alternifolia* Essential Oil against *Myzus persicae*

Talha Ali Chohan, Tahir Ali Chohan, Lijun Zhou, Qianqian Yang, Liao Min and Haiqun Cao

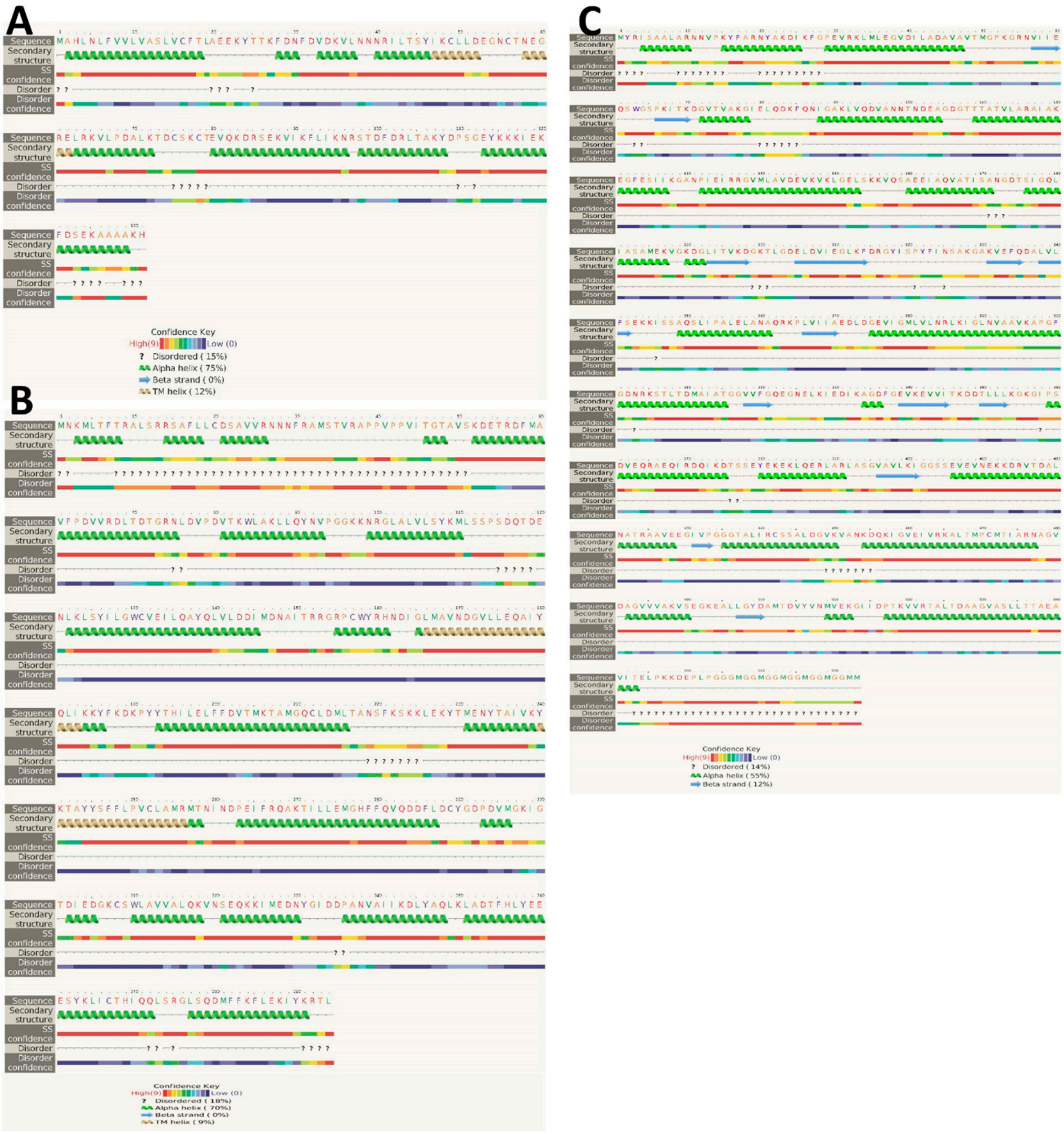

Figure S1. Secondary structures of (**A**) *OSD*, (**B**) *FPPS I* (**C**) *HSP 60* predicted with Phyre2 online webserver.

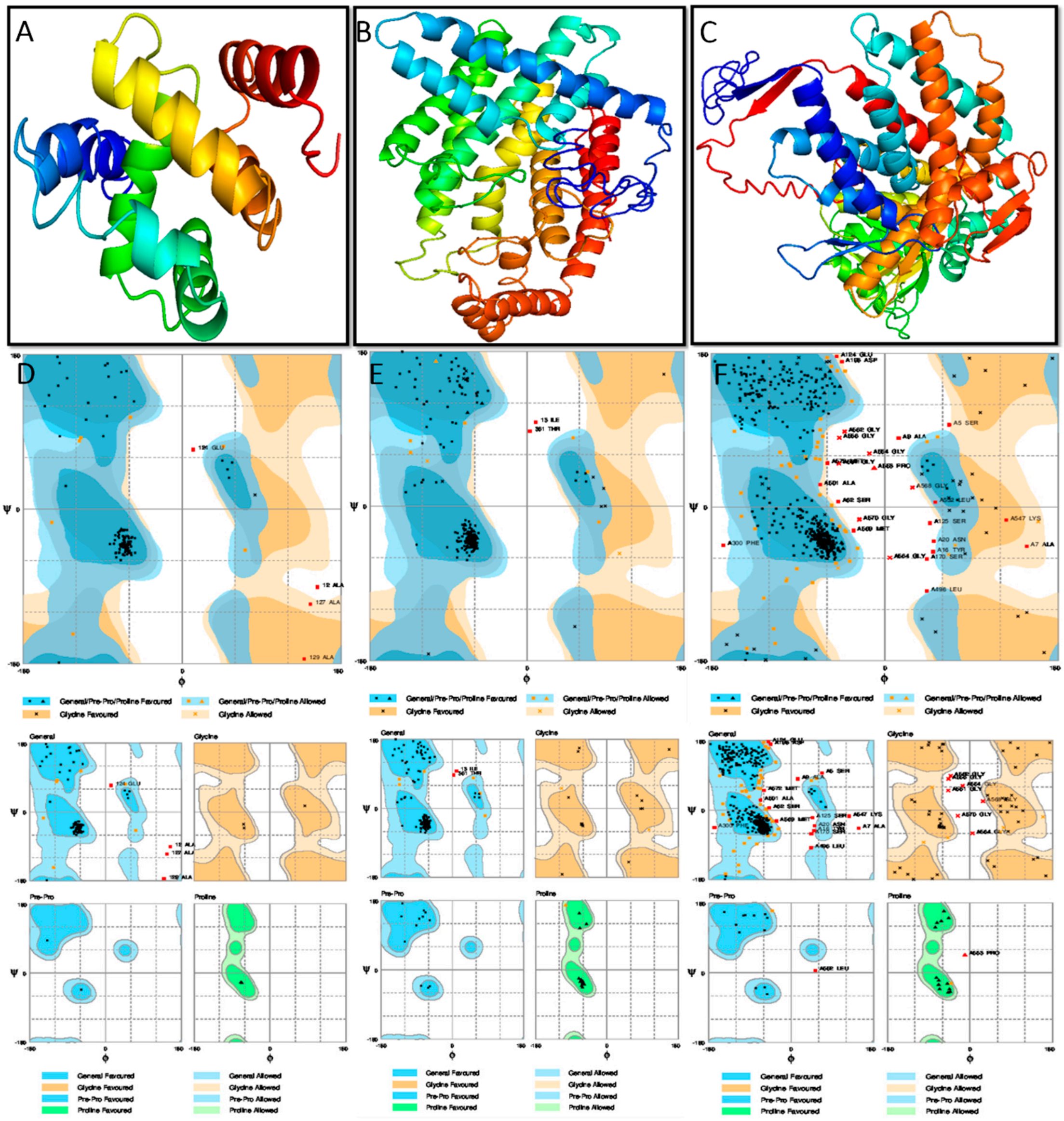

Figure S2. 3D structures of proteins built by I-TASSER. (**A**) *OSD*; (**B**) *FPPS I*; (**C**) *HSP 60*. Ramachandran Plot analysis performed with RAMPAGE online webserver. (**D**) *OSD*; (**E**) *FPPS I*; (**F**) *HSP 60*.

Table S1. List of the selected primers (forward and reverse) which used for the measurements of gene expression in the *M. persicae* at three different lethal concentration of the *M. alternifolia* oil.

Gene Name	Accession Number	Forward Primer	Reverse Primers	Reference	Gene Size
ANT	DQ407505	GCCGGTAATTTAGCATCAGG	CCTTGGACAAACAGTCTCCA	[1]	151
OSD	AJ634652	TCCCGAAGGAGCTGAACTTA	GCTTAGGGTCCCATTTGTCA	[1]	164
TOL	EB714328	AGCGCTTTCTGACGGAAATA	AGCATTCGAAGAAGCGATTG	[1]	177
HSP 60	EU334430	AGCATTGACCATGCCATGTA	AAACATCGGTCATTGCATCA	[1]	122
FPPS1	AJ250348	CGAACAGGCCATTTACCAGT	GACCCATCGCAGTTTTCATT	[1]	107
β-actin	[2]	GGTGTCTCACACACAGTGCC	CGGCGGTGGTGGTGAAGCTG	[2]	90–120
ACE	[2]	TAACGTAGTAGTGCCAAAGC	CACTGTAGAGCCATTAGCTG	[2]	90–120

Table S2. Summary of pre- and post-molecular modeling analysis.

Protein Name	Accession No	*β*-Sheet	*TM*-Helix	*α*-Helix	C-Score	Z-Score
OSD	CAI34909	0%	12%	75%	−0.73	2.82
FSSP I	XP_022183539	0%	9%	70%	−1.17	0.82
HSP 60	CAB58441	12%	—	55%	0.25	4.03

Table S3. Surflex score of docked ligands terpinen-4-ol and γ-terpinene for *OSD, FPPS I, and HPSA 60.*

Docking Complex	C Score [a]	Crash Score [b]	Polar Score [c]	G Score [d]	PMF Score [e]	D Score [f]	Chem Score [g]	Amino Acid Interaction
terpinen-4-ol-*OSD*	5.23	−0.47	0.55	−133.251	2.19	−60.908	−14.421	E62, LEU14, K65, R61, T18, N58 and V15.
γ-terpinene-*OSD*	2.22	−0.45	1.20	−138.868	2.054	−58.152	−13.986	V81, V33, R85, T26, LEU63, E59, E62 and LEU14.
terpinen-4-ol- *FPPS I*	4.55	−0.60	1.07	−133.295	−12.786	−50.833	−12.255	R 120, K266, M264, R121, G64, R360, F248 and R68.
γ-terpinene-*FPPS I*	2.55	−0.84	0.33	−136.064	−1.759	−51.141	−9.967	R68, K104, K66, K65, R121, R120 , M264, K266 and G265.
terpinen-4-ol-*HSP 60*	4.42	−0.44	1.39	−125.299	−0.846	−45.844	−12.697	T417, T74, D414, V73, D418, N101 and D71.
γ-terpinene-*HSP 60*	3.10	−0.56	0.44	−120.413	12.119	−56.424	−11.149	R424, E428, A425, N421, K519, E103 and A104.

[a] **CScore** is a consensus scoring which uses multiple types of scoring functions to rank the affinity of ligands, [b] **Crash-score** revealing the inappropriate penetration into the binding site, [c] **Polar** region of the ligand, [d] **G-score** showing hydrogen bonding, complex (ligand-protein), and internal (ligand-ligand) energies, [e] **PMF-score** indicating the Helmholtz free energies of interactions for protein-ligand atom pairs (Potential of Mean Force, PMF), [f] **D-score** for chRe and van der Waals interactions between the protein and the ligand, [g] **Chem-score** points for hydrogen bonding, lipophilic contact, and rotational entropy, along with an intercept term.

Table S4. Comparison between binding free energies of terpinen-4-ol bonded with *OSD, FPPS I, HSP 60*.

Protein-Inhibitor	OSD-Terpinen-4-ol	FPPS I-Terpinen-4-ol	HSP 60-Terpinen-4-ol
ΔE_{vdW} [a]	−16.00	−18.12	−16.83
ΔE_{ele} [a]	−14.59	−7.31	−8.59
$\Delta G_{nonpol,\ sol}$ [a]	−2.45	−2.48	−2.24
$\Delta G_{ele,\ sol\ (PB)}$ [a]	23.28	19.84	20.76
$\Delta G_{ele,\ sol\ (GB)}$ [a]	22.70	18.49	19.73
$\Delta E_{vdW}+\Delta G_{nonpol,sol}$ [a]	−18.45	−20.60	−25.42
$\Delta E_{ele}+\Delta G_{ele,sol\ (PB)}$ [a]	8.69	12.53	12.17
$\Delta E_{ele}+\Delta G_{ele,sol\ (GB)}$ [a]	8.11	11.18	11.14
$\Delta G_{pred\ (PB)}$ [b]	−9.08	−7.51	−6.68
$\Delta G_{pred\ (GB)}$ [b]	−10.34	−9.43	−7.94

[a] All energies are in kcal/mol, ΔH: the enthalpy changes, $\Delta H = \Delta G_{ele} + \Delta G_{vdW} + \Delta G_{nonpol,\ sol} + \Delta G_{ele,\ sol}$,[b] ΔG_{pred}: the calculated binding free energy by MMPB(GB)SA method.

Reference

1. Ayyanath, M.-M.; Cutler, G.C.; Scott-Dupree, C.D.; Prithiviraj, B.; Kandasamy, S.; Prithiviraj, K. Gene expression during imidacloprid-induced hormesis in green peach aphid. *Dose-Response* **2014**, *12*, doi: 10.2203/dose-response.13-057.Cutler.
2. Puinean, A.M.; Foster, S.P.; Oliphant, L.; Denholm, I.; Field, L.M.; Millar, N.S.; Williamson, M.S.; Bass, C., Amplification of a cytochrome P450 gene is associated with resistance to neonicotinoid insecticides in the aphid *Myzus persicae*. *PLOS Genet.* **2010**, *6*, e1000999.
